# ctDNA Concentration, MIKI67 Mutations and Hyper-Progressive Disease Related Gene Mutations Are Prognostic Markers for Camrelizumab and Apatinib Combined Multiline Treatment in Advanced NSCLC

**DOI:** 10.3389/fonc.2020.01706

**Published:** 2020-09-04

**Authors:** Yao Chen, Xiaobin Li, Guifeng Liu, Shifu Chen, Mingyan Xu, Lele Song, Yina Wang

**Affiliations:** ^1^Department of Oncology, First Affiliated Hospital, College of Medicine, Zhejiang University, Hangzhou, China; ^2^HaploX Biotechnology, Shenzhen, China; ^3^Department of Radiotherapy, The Eighth Medical Center of the Chinese PLA General Hospital, Beijing, China

**Keywords:** NSCLC, immunotherapy, PD-1, angiogenic, ctDNA, TMB, PFS, Apatinib

## Abstract

Immunotherapy by immune checkpoint inhibitors (ICIs) has showed outstanding efficacy in the treatment of advanced non-small cell lung cancer (NSCLC). The combination of immunotherapy with anti-angiogenic therapy exhibited enhanced efficacy in multiline treatment. However, the potential biomarkers for predicting and monitoring the therapeutic response of the combined therapy remain undefined. In this study, we performed a pilot study by prospectively recruiting 22 advanced NSCLC patients who failed to previous lines of chemotherapy, chemoradiotherapy, TKI therapy, surgery, or any combination of the therapies, and investigated the prognostic factors for patients who received anti-PD-1 (Camrelizumab) and anti-angiogenic (Apatinib) combined therapy. The objective response rate (ORR) assessed by an independent radiology review was 22.7%, and the median progression-free survival (PFS) was 5.25 months. We found that high concentration of circulating-free DNA (cfDNA) (HR = 27.75, *P* = 0.003), MIKI67 mutation (HR = 114.11, *P* = 0.009) and gene variations related to hyper-progressive disease (HPD) (HR = 36.85, *P* = 0.004) were independent risk factors and exhibited significant correlation with PFS. Circulating tumor DNA (ctDNA) mutational status was also a predicting indicator for PFS. In contrast, the blood tumor mutational burden (bTMB) could not stratify the clinical benefit in this combined therapy (HR = 0.81, *P* = 0.137). Furthermore, we found that the variant allele fraction (VAF) of mutations in ctDNA was sensitive indicators of therapeutic response and therefore can be used to monitor the tumor relief or progression. In conclusion, cfDNA concentration, MIKI67 mutations and HPD-related mutations were independent risk factors and PFS predictors for multiline combined anti-angiogenic/ICI combined therapy. ctDNA may be a novel monitoring biomarker for therapeutic response and predicting biomarker for prognosis in future combined therapy involving PD-1 blockade.

## Introduction

Immune checkpoint inhibitors (ICIs), such as programmed death 1 (PD-1)/programmed death ligand 1 (PD-L1) inhibitors have made a significant breakthrough in lung cancer treatment (1–3). Antibody blockade of the PD pathway rectifies immunodeficiency in tumor microenvironment, and empowers CD8+ T cells with the capability to kill tumor cells efficiently (4, 5). Along with success in clinical trials of immunotherapy, many more therapeutic options have appeared for lung cancer patients (6–12).

In patients with previously treated lung cancer, especially patients with wild-type driver genes, ICIs have substantially improved clinical prognosis compared with chemotherapy, as evidenced by an improvement of overall survival (OS) from 9.6 to 13.8 months in OAK cohorts (13). However, the achieved objective response rate (ORR) was less than 20% for anti-PD-1/PD-L1 antibody monotherapy in second-line or higher settings, including Nivolumab, Pembrolizumab, and Atezolizumab (6, 13, 14). Therefore, there is an urgent need to explore appropriate methods for improving the efficacy of immunotherapy and selecting patients potentially benefit from immunotherapy at multiline levels.

Many studies have focused on combination strategies to improve clinical efficacy of ICIs. The combination of Atezolizumab with Bevacizumab, Carboplatin, and Paclitaxel (ABCP) had significantly improved OS (19.2 months vs. 14.7 months) and PFS (8.3 months vs. 6.8 months) for metastatic non-squamous non-small cell lung cancer (NSCLC) in the phase III IMpower150 trial (9). Evidence has also suggested that anti-PD-1 and anti-vascular endothelial growth factor (VEGF) combined therapy may be a more favorable treatment option than any single reagent for NSCLC patients who had failed on the first-line or later treatment, and this therapeutic response was not affected by VEGF mutational status (15–17). CheckMate 227, another phase III trial in advanced NSCLC, suggested that Nivolumab plus Ipilimumab resulted in a longer OS independent of the PD-L1 expression level (18). This could be because the differential immune effects of CTLA-4 vs. PD-1 inhibition recruited effective antitumor immunity from the peripheral compartment, which is increasingly recognized as an important mechanism of response to immunotherapy (19–21). The combination of Pembrolizumab with chemotherapy exhibited superior ORR (57.9% vs. 38.4%), OS (15.9 months vs. 11.3 months) and PFS (6.4 months vs. 4.8 months) compared with placebo group for previously untreated metastatic, squamous NSCLC, demonstrated by the phase III KEYNOTE-407 trial (22). Similarly, in patients with treatment-naïve metastatic non-squamous NSCLC without EGFR or ALK mutations, the combination of standard chemotherapy with Pembrolizumab resulted in significantly longer OS (1-year OS rate: 69.2% vs. 49.4%) and PFS (8.8 months vs. 4.9 months) compared with placebo in KEYNOTE-189 trial (11). Moreover, PEMBRO-RT, a multicenter, randomized phase II study for metastatic NSCLC patients after chemotherapy failure, indicated that the ORR at 12 weeks was improved from 18 to 36% when radiotherapy was combined with Pembrolizumab, although the improvement did not reach statistical significance (*P* = 0.07) (23).

All the above clinical trials suggest that immunotherapy combined with chemotherapy or radiotherapy improved clinical efficacy in NSCLC patients. However, most randomized phase III clinical trials using combined immunotherapy were designed for first-line therapy, and there are few data for NSCLC patients in second-line or higher settings. In this study, we therefore performed a pilot study by recruiting a total of 22 NSCLC patients who failed to previous lines of chemotherapy, chemoradiotherapy, TKI therapy, surgery, or any combination of the therapies, and investigated the clinical efficacy of Camrelizumab, a humanized, high-affinity IgG4-kappa mAb against PD-1, combined with Apatinib, a small molecular drug targeting vascular endothelial growth factor receptor-2 (VEGFR-2). Since the traditional biomarkers for immunotherapy, such as the PD-L1 expression and tumor mutation burden (TMB) appeared to have limited predicting values in several clinical trials using combined immunotherapy (11, 12, 22, 24, 25), we investigated the values of a few new biomarkers in predicting and monitoring the therapeutic response and prognosis in second-line or higher settings when ICI was combined with anti-angiogenic therapy.

## Materials and Methods

### Ethic Approval by Participating Hospitals

All study plans and protocols for the study were submitted to the ethics/licensing committee of the First Affiliated Hospital of Zhejiang University for review and approval before the start of the study, and were approved by the corresponding committee. Confirmation of approval for clinical study was received from the ethics board (approval number: 2018-775-1) before the start of the study. All experiments, methods, procedures and personnel training were carried out in accordance with relevant guidelines and regulations of participating hospitals and laboratories.

### Study Design, Patients, and Samples

Patients recruited in this study were advanced NSCLC patients who received at least one line of treatment (chemotherapy or target therapy) and exhibited disease progression, and were recommended for subsequent therapy using Camrelizumab (or SHR-1210, Hengrui Medicine, Jiangsu, China) (200 mg q2w) combined with Apatinib (Hengrui Medicine, Jiangsu, China) (250 mg qd). Blood samples were collected at the First Affiliated Hospital of Zhejiang University. All patients received written informed consent for the use of clinical samples. Patient information was kept anonymous for confidentiality.

A total of 22 advanced lung cancer patients were recruited in this study between July 1, 2018 and October 31, 2019. The baseline characteristics of these patients are described in Table 1, and the detailed information has been included in Table 2, Supplementary Tables S2, S3. The median age of these patients was 61.5 years old (33∼73 years old). Seventeen patients (77.3%) were male and five (22.7%) were female, and all the female patients (*n* = 5) were non-smokers, whereas 15 (88.2%) male patients had more than 20 pack years of smoking history. The number of lung adenocarcinoma patients (*n* = 10) was approximately equal to the patients harboring squamous cell carcinoma (*n* = 12). Seventeen patients (77.3%) were diagnosed with stage IV lung cancer, and five patients were at stage IIIB. Fourteen patients (63.6%) underwent this combined immunotherapy as second-line therapy, and the remaining eight patients (36.4%) received this therapy as third-line or higher treatments. The clinical information of all patients is summarized in Table 1. Disease control rate (DCR) and progression free survival (PFS) was used to assess the response of therapy. The expression of PD-L1 was determined before the first-line therapy on primary tumors and not on recurrent or resistant tumors, and immunohistochemistry was performed with Dako PDl-L1 IHC 22C3 pharmDx [Agilent Technology (China) Co., Ltd.] using the 22C3 antibody.

**TABLE 1:** Patient characteristics grouped by clinicopathological factors.

Clinicopathological factors	Number of patients	Percentage (%)
Total	22	100
Age		
<40	1	4.5
40–49	2	9.1
50–59	3	13.6
60–69	12	54.6
≥70	4	18.2
Gender		
Male	17	77.3
Female	5	22.7
Pathological types		
ADC	10	45.5
SCC	12	54.5
Smoking history		
Yes	15	68.2
No	7	31.8
Stage		
IIIB	5	22.7
IV	17	77.3
Lines of therapy		
2nd	14	63.6
3rd	6	27.3
4th–6th	2	9.1

*ADC, adenocarcinoma; SCC, squamous cell carcinoma.*

**TABLE 2:** The relationship between therapeutic responses and clinicopathological factors.

Clinicopathological factors	DCR	PD	*P*-value
Gender			
Male	11	6	0.61*
Female	2	3	
Histology			
ADC	5	5	0.67*
SCC	8	4	
Smoking history			
Ever	10	5	0.38*
Never	3	4	
Stage			
IIIB	3	2	1*
IV	10	7	
Mutational status in ctDNA			
Negative	7	0	0.017*
Positive	6	9	
bTMB			
High	4	4	0.66*
Low	9	5	
Lines of therapy			
2nd line	9	5	0.66*
3rd–6th line	4	4	
PFS (mean, 95% CI)			
2nd line	9.03 (6.03∼12.02)	3.19 (1.50∼4.88)	0.010^#^
3rd–6th line	7.83 (0.62∼15.03)	2.10 (0.17∼4.13)	0.088^#^
TKI-related driver gene status			
With mutations	1	3	0.26*
Without mutations	12	6	

*DCR, disease control rate; PD, progressed disease; ADC, adenocarcinoma; SCC, squamous cell carcinoma; ctDNA, circulating tumor DNA; bTMB, blood tumor mutational burden. ^∗^Calibrated Chi-square test or Fisher exact probability test; ^#^unpaired *t*-test.*

### Sample Preparation, Library Construction, Targeted Sequencing, and Data Processing

Blood samples from patients were collected in Ethylene Diamine Tetraacetic Acid (EDTA) tubes and centrifuged at 1600 *g* for 10 min and at 4°C. The supernatants were further centrifuged at 10,000 × *g* for 10 min at 4°C, and plasma was harvested and stored at −80°C until further use. Circulating tumor DNA (ctDNA) was extracted from 3 to 3.5 ml plasma using the QIAamp Circulating Nucleic Acid kit (Qiagen, Inc., Valencia, CA, United States) according to the manufacturer’s instructions. Blood cell fragments (including peripheral blood lymphocytes and red cells) were preserved at −20°C for further study. We applied the RelaxGene blood DNA system (Tiangen Biotech) to extract genomic DNA from peripheral blood lymphocytes (PBLs) as the normal control for mutation calling from cancer tissues and ctDNA. DNA was quantified with the Qubit 2.0 Fluorometer and the Qubit dsDNA HS assay kit (Thermo Fisher Scientific, Inc., Waltham, MA, United States) according to manufacturer’s instructions. Fragmented genomic DNA underwent end-repairing, A-tailing and ligation with indexed adapters sequentially, followed by size selection using Agencourt AMPure XP beads (Beckman Coulter Inc., Brea, CA, United States), and DNA fragments were used for library construction using the KAPA Library Preparation kit (Kapa Biosystems, Inc., Wilmington, MA, United States) according to the manufacturer’s protocol. Hybridization-based target enrichment was carried out with HaploX pan-cancer gene panel (605 cancer-relevant genes, HaploX Biotechnology, gene list is provided in Supplementary Table S1) for cancer tissue sequencing. Seven to eight polymerase chain reaction (PCR) cycles, depending on the amount of DNA used, were performed by pre-capture ligation-mediated PCR (Pre-LM-PCR) Oligos (Kapa Biosystems, Inc.) in 50 μl reactions. ctDNA sequencing was then performed on the Illumina Novaseq 6000 system according to the manufacturer’s recommendations at an average depth of 20,000×.

Data which meet the following criteria were chosen for subsequent analysis: the ratio of remaining data filtered by fastq in raw data is ≥85%; the proportion of Q30 bases is ≥85%; the ratio of reads on the reference genome is ≥85%; target region coverage ≥98%; average effective sequencing depth in ctDNA is ≥ 3000×. The called somatic variants need to meet the following criteria: the read depth at a position is ≥20×; the variant allele fraction (VAF) is ≥0.5% for ctDNA and ≥2% for PBL genomic DNA; somatic-*P*-value ≤0.01; strand filter ≥1. VAF were calculated for Q30 bases. The copy number variation (CNVs) was detected by CNVkit version 0.9.3^1^. Further analyses of genomic alterations were also performed, including single nucleotide variants (SNVs), CNVs, insertion/deletion (Indels), fusions, and structural variation.

### Statistical Analysis

Statistical analysis was performed and figures were plotted with Graphpad Prism 5.0 software (GraphPad Software, Inc., La Jolla, CA, United States). Student *t*-test was performed when two groups were compared, and ANOVA and *post hoc* tests were performed when three or more groups were compared. Chi-square test, calibrated Chi-square test or Fisher exact probability test were performed when rate or percentage was compared for significance. Figures for mutation spectrum were made with the R software^2^. Univariate and multivariate analyses were performed using the SPSS 17.0 software (IBM China Company Limited, Beijing, China). *P* < 0.05 was regarded as statistically significant.

## Results

### Genetic Alterations Were Capable of Predicting the Therapeutic Response and Prognosis of the Multiline Anti-angiogenic/ICI Combined Therapy

Twenty-two patients involved in this study received Camrelizumab combined with Apatinib therapy (Table 1). Fourteen patients received the combined treatment as the second-line therapy, while the rest as the third to sixth line therapy. The ORR and DCR assessed by an independent radiology review were 28.6% (4/14) and 64.3% (9/14), respectively, for the second-line therapy, and were 12.5% (1/8) and 50.0% (4/8), respectively, for the third to sixth line therapy (Table 2). All patients were followed up until disease progression (PD) or the end of this study. The progression-free survival (PFS) for all patients ranged from 1.4 to 15.5 months (median at 5.25 months). Data in Table 2 showed a significantly longer PFS in patients with DCR than those with PD at the second-line therapy (*P* = 0.010, unpaired *t*-test), and similar trend can also be observed with patients at third to sixth line therapy (*P* = 0.088, unpaired *t*-test), while no significant difference was observed in PFS between patients with second-line therapy and patients with third to sixth line therapy (Table 2). We stratified all patients into the ADC group and SCC group, and examined the potential correlation between the above factors and patients’ response (DCR or PD). It can be observed from Supplementary Table S2 that the significant difference in PFS between patients with DCR and PD can be observed in SCC patients at second-line level (*P* = 0.04), while a trend of better PFS was also observed with ADC at second-line level (*P* = 0.15).

**FIGURE 1:**
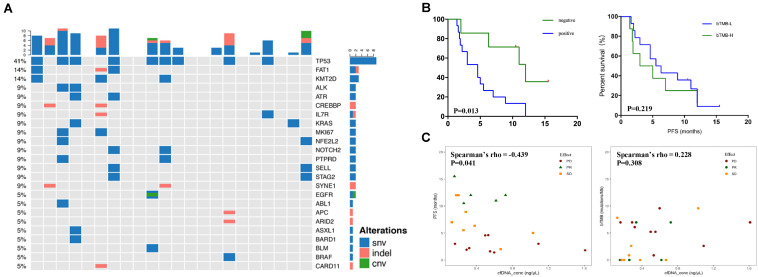
The relationship between blood mutational landscape and therapeutic response. **(A)** The pre-therapeutic blood ctDNA mutational landscape. **(B)** Keplan–Meier survival analysis of patients with negative or positive ctDNA mutation detection (left panel, 7 patients with negative and 15 patients with positive ctDNA mutation detection) or patients with low or high bTMB levels (right panel, 8 patients with high and 14 patients with low bTMB levels). **(C)** Correlation between cfDNA concentration and PFS (left panel) or bTMB (right panel).

The mutational spectrum of circulating-free DNA (cfDNA) was established using the pre-therapeutic blood samples (Figure 1A). Mutations were detected in cfDNA of 15 patients while not detected in 7 patients. The NGS sequencing yielded a total of 80 somatic SNVs, 19 small insertions/deletions (indels), and 4 CNVs. TP53 (41%), FAT1 (14%), and KMT2D (14%) exhibited the highest mutational frequencies (Figure 1A). Few patients carried TKI-related driver gene mutations, including EGFR (one patient), BRAF (one patient), PIK3CA (one patient), and ERBB2 (one patient).

We further explored the potential predicting efficacy of genetic variations on PFS and therapeutic response (Figures 1B,1C). Patients with PR and SD were combined into DCR group due to the limited number of patients. It was found that patients with no detectable ctDNA mutations (ctDNA-negative group) showed significantly better PFS than patients with ctDNA mutations (ctDNA-positive group) (*P* = 0.017, Fisher exact probability test), suggesting that the pre-therapeutic ctDNA mutations were predictive for PFS (Figure 1B and Table 2). Further analysis on ADC and SCC individually revealed that the significant correlation between ctDNA mutational status and response was mainly reflected in ADC (*P* = 0.008, Supplementary Table S2). In contrast, the pre-therapeutic blood TMB (bTMB) level was not able to predict the PFS, when the 66.7 percentile bTMB value (6.96 mutations/Mb) was used to discriminate TMB-high (TMB-H) from TMB-low (TMB-L) (Figure 1B, Table 2 and Supplementary Table S3). This observation indicates that the predictive capability of bTMB may be compromised in the second-line or multiline combined immunotherapy. Further correlation analysis showed that PFS had significant correlation with the blood cfDNA concentration (Spearman’s rho = −0.439, *P* = 0.041), in which patients with better therapeutic response (PR or SD) correlated with longer PFS and lower cfDNA concentration, while no significant correlation can be found between bTMB and blood cfDNA concentration (Figure 1C and Supplementary Table S3). Furthermore, it appeared that gender, pathological types, smoking history, cancer stage, line of therapy, and the presence of TKI-related driver gene mutations did not affect the therapeutic response (DCR or PD) (Table 2). The correlation between PD-L1 expression and PFS (Supplementary Figure S1A), bTMB (Supplementary Figure S1B), ctDNA concentration (Supplementary Figure S1C), and response (Supplementary Figure S1D) were investigated, and no clear correlation was identified. In addition, PD-L1 expression levels, whether grouped by positive or negative expression (*P* = 0.18) or by 50% threshold (*P* = 0.39), did not show significant stratification on response (Supplementary Figures S1E,F).

**FIGURE 2:**
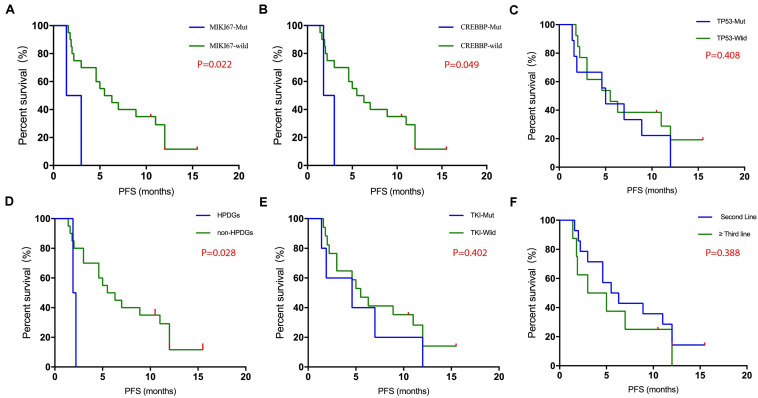
Keplan–Meier survival analysis of various mutational status. The mutational status of MIKI67 (**A**, 2 muts and 20 WTs), CREBBP (**B**, 2 muts and 20 WTs), TP53 (**C**, 12 muts and 10 WTs), HPD genes (**D**, 2 with HPDG alterations and 20 without), TKI-relevant mutations (**E**, 4 with TKI-related mutations and 18 without), and lines of therapy (**F**, 12 at second-line and 10 at higher lines) were compared and shown as indicated.

The relationship between gene variations and PFS was investigated in detail. Kaplan–Meier survival analysis showed that patients with MIKI67 mutations (Log-rank test *P* = 0.022) or CREBBP mutations (Log-rank test *P* = 0.049) exhibited significantly worse PFS than those without mutations (Figures 2A,B). In contrast, no such difference was observed in patients with or without TP53 mutations (Figure 2C). Interestingly, we found that patients who carried mutations in genes related to hyper-progressive diseases (HPDs) (one patient carried EGFR amplification and one patient carried FGF4 amplification) showed significantly worse PFS compared with those without such mutations (Log-rank test *P* = 0.028) (Figure 2D), while no significant difference in PFS was found between patients with or without TKI-related driver gene mutations (Figure 2E). Finally, no significant difference was found between patients with second-line therapy and those with third- to sixth-line therapy (Figure 2F and Table 2).

**FIGURE 3:**
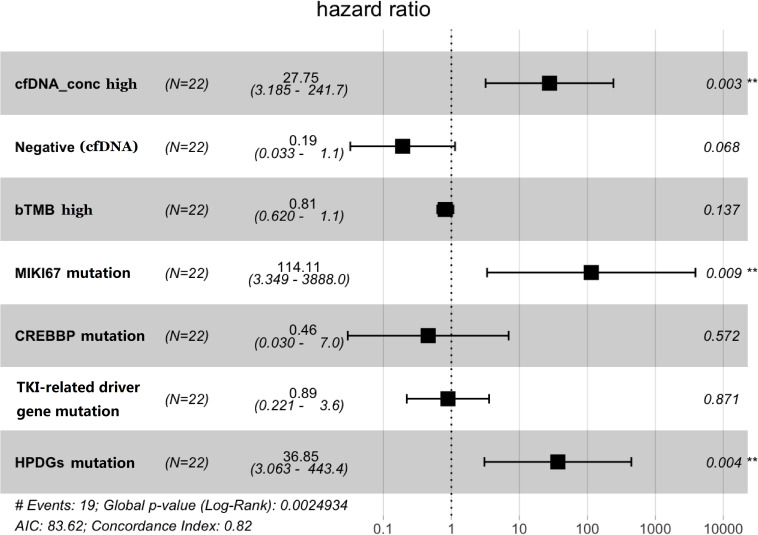
The results of multivariate Cox regression analysis for various potential risk factors. cfDNA concentration (*P* = 0.003), MIKI67 mutations (*P* = 0.009), and HPD gene mutations (*P* = 0.004) were identified as independent risk factors, while a trend of significance can be observed with ctDNA mutations (*P* = 0.068). Negative (cfDNA) indicates that no mutations in any gene were found in the 605 gene-panel test.

Multivariate Cox regression analysis (Figure 3) was performed using variables with *P* ≤ 0.3 as described above (Figures 1B,C, 2). The concentration of cfDNA was found to be an independent predictor of PFS (HR = 27.75, *P* = 0.003), in which higher cfDNA concentration correlated with poorer outcomes (Figure 3). Similarly, patients harboring mutations in MIKI67 (HR = 114, *P* = 0.009) or genes relating to HPDs (HR = 36.85, *P* = 0.004) were also significantly associated with shorter PFS. Meanwhile, due to the limited number of patients, the ctDNA-negative group did not show more benefit from this combined immunotherapy compared with the ctDNA-positive group, although a trend of difference was observed (HR = 0.19, *P* = 0.068). As expected, bTMB could not predict the clinical benefits in this combined therapy.

### ctDNA Is Capable of Monitoring the Therapeutic Responses of the Multiline Anti-angiogenic/ICI Combined Therapy

**FIGURE 4:**
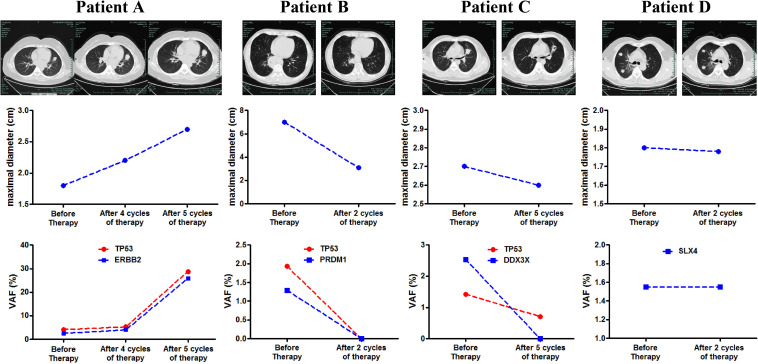
Correlation between tumor diameter change and ctDNA mutational change following the multiline anti-angiogenic/ICI combined therapy. The CT images, tumor diameter changes and VAF changes of blood ctDNA mutations of four patients **(A–D)** were illustrated.

We further investigated the potential of blood ctDNA in therapeutic response monitoring. Plasma samples were obtained before and after treatment at key assessment points of therapeutic response in parallel with imaging examination. Figure 4 shows several examples of patients with distinct therapeutic responses. We found that the changes in variant allelic frequency (VAF) of mutations exhibited identical trend to the changes in target tumor maximal diameter, whether in progressed disease (patient A), partial response (patient B), or in stable disease (patient D). More interestingly, we observed that the VAF of ctDNA decreased significantly in one patients following combined immunotherapy without significant change in tumor size (patient C), but exhibited and a clear cavity inside the tumor. The patient was progression free for 12 months and achieved PR ultimately. These results indicate that dynamics of ctDNA may be more sensitive than imaging examination to monitor the therapeutic response to combined immunotherapy. Furthermore, the regression tree analysis has been performed. PFS, cfDNA concentration, ctDNA detection (negative or positive), bTMB, MIKI67 mutation, CREBBP mutation, TKI-related driver gene mutation, HPDG mutation, TP53 mutation, and lines of therapy were set as the independent variable and the patient risk (determined by the significant factors in Figure 3) was set as the dependent variable. Although ctDNA detection appeared to be the only significant factor that may predict the risk of patients, the test was not conclusive as the number of patients involved was limited.

## Discussion

In this study, we prospectively enrolled 22 patients with advanced NSCLC and studied the therapeutic responses and prognostic factors of the combined anti-angiogenic/ICI therapy at second-line and multiline levels. Previous research reported that antiangiogenic drugs can exert immune activation by inhibiting VEGF, promoting dendritic cell maturation, increasing T cell infiltration, and reprogramming the immune microenvironment (26). Therefore, anti-vascular drugs may reverse VEGF-mediated immunosuppression, thereby enhancing the antitumor activity of immunotherapy. Compared with the therapeutic responses of second- or multi-line PD-1 blockade previously reported (13, 14), PD-1 blockade combined with anti-angiogenic drugs had obvious clinical advantages, significantly improving ORR and PFS. This combined therapy could have better ORR, DCR, and PFS in the second-line treatment than the multiline treatment. In this study, we found a trend that the PFS of patients with second-line therapy may be better than that of the 3rd—6th lines of therapy, which supported previous observations. We also found that cfDNA concentration, MIKI67 mutations, and HPD-related gene mutations and potentially ctDNA mutations, were independent risk factors for therapeutic response prediction, while age, gender, lung cancer subtypes, smoking history, and bTMB did not affect the response.

Previous studies have shown that ctDNA was able to predict PFS in the first-line or multi-line immunotherapy of NSCLC (1, 27, 28). Similar capability were confirmed in colorectal cancer (29, 30), breast cancer (31, 32), liver cancer (33), and gastric cancer (34, 35). In this study, we found that ctDNA was also capable of predicting the response of NSCLC to multiline anti-angiogenic/ICI combined therapy. Patients with positive ctDNA exhibited worse prognosis (PFS) than patients with negative ctDNA. This may be due to fact that higher tumor load in ctDNA-positive patients led to poor treatment response and poor tumor remission. It can be concluded from previous and our studies that the survival of ctDNA-positive patients will be worse than ctDNA-negative patients, regardless of single-agent or combined immunotherapy, and regardless of first-line or multi-line therapy. In addition, we also found that the VAF of ctDNA mutations was a good indicator of response of the combined therapy. In this study, prospective collections of blood samples before and after treatment and tests using NGS panel allowed the tracking of multiple genes with fluctuating VAF found in ctDNA. High concordance was found between the change of VAF and the tumor size. Due to the limited number of mutations that can be detected in ctDNA, our observation suggested that targeting a few mutations with high VAF in ctDNA may be sufficient to monitor the therapeutic response. We also found that ctDNA may be more sensitive and have predicting efficacy compared with imaging studies. This is supported by previous reports showing that ctDNA can predict tumor recurrence or remission much earlier than imaging examination (36), which is important for decision-making in therapeutic strategy selection. Therefore, we speculate that if ctDNA is used in combination with imaging, they can better monitor and predict the response and prognosis of patients than using one method alone. In addition, we found that cfDNA concentration was significantly correlated with PFS and was an independent risk factor for patients, suggesting that the plasma cfDNA level can predict the response and prognosis regardless of the mutational status, which was also supported by previous reports (37–39).

Many studies support the viewpoint that bTMB can be used as a biomarker to predict clinical efficacy in anti-PD-1/PD-L1 immunotherapy. Patients with high bTMB have a better ORR and PFS than patients with low bTMB (1, 40). However, a retrospective analysis of the POPLAR and OAK studies found that bTMB may not effectively distinguish the benefit in OS of patients receiving immunotherapy at second-line or higher (1, 41). Giles et al. (42) recently also found worse PFS and OS in NSCLC patients with higher bTMB in first-line immunotherapy. This suggests us that evidence is still needed to support the effectiveness of bTMB as a biomarker in immunotherapy, especially in multiline combined therapy. We provided such evidence in this article and found that the level of bTMB cannot effectively distinguish the benefit population in second-line and multi-line anti-angiogenic/ICI combined therapy. We speculate that one of the possible reasons is that the combined therapy improved the patients’ response, and weakened the stratification of bTMB on the patients’ response and prognosis observed in single-agent based immunotherapy.

It has been previously reported that PD-L1 expression can effectively stratify the therapeutic effect of ICIs, and the PD-L1 expression has been approved by the FDA as a companion diagnostic marker for anti-PD-1/PD-L1 treatment. Patients with high PD-L1 expression are more likely to benefit from immunotherapy (43). However, the PEMBRO-RT study found that PFS and OS in patients with PD-L1 high expression were shorter in the second-line immunotherapy combined with radiotherapy for lung cancer (23), also suggesting that the efficacy of biomarkers in multiline combined therapy involving PD1/PD-L1 blockade may be different from previous observations with immunotherapy alone. Ideally, the PD-L1 expression status of all patients with recurrent or resistant tumors should be reexamined, and multiline therapeutic strategies should be established based on the information. However, practically, many patients were reluctant to receive a second biopsy with both PD-L1 test and NGS test, because they believe that immunotherapy combined with other therapies was possibly the only option for them when the disease progressed, and therefore there was no need to repeat the tests anymore. As a result, the reexamination rate in these patients was very low. We were facing the similar situation in this study. Therefore, we tried to link the PD-L1 expression from primary tumor with the response of 2nd–6th lines therapy to see if we can identify any correlation, while no significant correlation was found, as shown in Supplementary Figure S1. Another concern is that due to the better response in patients with combined therapy, the effect of stratification by PD-L1 may not be significant anymore, and patients with negative PD-L1 expression may also benefit from the therapy, which was reflected in some recent studies (15–17).

Previous reports have shown that tumors were not effectively controlled and hyperprogression finally developed after immunotherapy in some patients (44). Further clinical studies have shown that these patients may carry genetic alterations, such as EGFR amplification, MDM2/MDM4 amplification, and chromosome 11 band 13-related gene amplification (FGF4, FGF19) (45). In this study, we prospectively enrolled two patients, who carried EGFR amplification and FGF4 amplification, respectively. They quickly developed PD with PFS of 1.9 and 2.2 months, respectively, far lower than those who did not carry hyperprogression-relevant gene mutations. Our observation suggests that the clinical response of patients with hyperprogression-relevant gene mutations was also poor in multiline anti-agiogenic/ICI combined therapy. Similarly, evidence also showed that NSCLC patients with TKI-related driver gene mutations often had poor ORR and PFS in anti-PD-1/PD-L1 treatment, in which the evidence for EGFR mutations and ALK fusions were strong, while other TKI-related driver gene mutations were not confirmed (46, 47). In this study, we compared patients with or without TKI-related driver gene mutations and found no significant differences in PFS between the two groups, suggesting that not all TKI-related driver gene mutations affect anti-angiogenic/ICI combined therapy. Interestingly, we found that two patients with ALK mutations (not fusions) with PFS of 12 and 1.4 months, respectively. The latter had a shorter PFS possibly due to a MIKI67 mutation, since MIKI67 mutations were an independent factor of poor prognosis. Our observation suggests that ALK non-fusion mutations may not affect the response of the combined therapy.

There were some limitations of this study. Firstly, heterogeneity of therapy was a weakness of the study, especially when therapeutic lines from 2nd to 6th were all involved. This is why we stratified the patients into 2nd line and 3rd–6th lines in this study, at least the therapy for 2nd line patients was homogeneous. By comparing these two populations, we found that the PFS of 2nd line treatment might be better than that of 3rd—6th treatment (Supplementary Table S2), which was one of main findings in the study. Secondly, two control cohorts, including one Camrelizumab and one Apatinib cohort, should be included to compare with the Camrelizumab and Apatinib combined cohort. However, it is difficult in reality to recruit patients using Camrelizumab or Apatinib alone, since both doctors and patients are aware that patients with combined therapy may potentially respond better than those treated with a single drug, therefore, challenges exist in both practical therapy and ethics if single drug group is recruited in parallel. However, some previous patients using the single drug may be involved as the control cohort. Thirdly, it was unfortunate in this study that we were unable to obtain any tissue samples from patients at advanced therapy stages, and therefore the test for tissue TMB was not possible. This was mainly because patients normally receive the tissue sampling and test when they are first diagnosed (before any therapy), and tissue sampling at later treatment is performed only when specific genetic alterations related to disease progression are highly suspected. In future study, tissue TMB should be obtained whenever possible, since difference might exist between tissue and blood TMB in therapy guidance. Fourthly, a relatively small cohort was one limitation of the study. For example, Figure 2 showed all mutations with significant stratification of survival. Due to the limited number of patients with MIKI67 or CREBBP mutants, the conclusion was not solid, although significant stratification was observed. The results need further validation with larger cohort in future.

## Conclusion

In summary, we found that the combination of anti-angiogenic with PD-1 blockade therapy significantly enhanced the ORR and PFS of NSCLC patients in second-line or multiline treatment. ctDNA concentration, MIKI67 mutation, and HPD-relevant gene mutations were independent risk factors for PFS. The blood ctDNA mutations can potentially identify the benefit population and predict the patients’ response and prognosis. In addition, ctDNA detection at series time points can effectively monitor disease relief or progression. Our study provided valuable evidence for treatment strategy selection in the second-line and multiline anti-angiogenic/ICI combined treatment of advanced NSCLC. There were also limitations in this study, mainly due to the limited number of patients included, while more patients should be enrolled in the future to confirm the conclusion of this study.

## Data Availability Statement

The datasets presented in this study can be found in online repositories. The names of the repository/repositories and accession number(s) can be found below: Genome Sequence Archive (https://bigd.big.ac.cn/gsa-human/browse/HRA000239).

## Ethics Statement

The studies involving human participants were reviewed and approved by The First Affiliated Hospital of Zhejing University. The patients/participants provided their written informed consent to participate in this study. Written informed consent was obtained from the individual(s) for the publication of any potentially identifiable images or data included in this article.

## Author Contributions

YW and LS designed the study and proofread the manuscript. YC and XL provided the samples and collected the clinical information and diagnostic information. YC, XL, and LS performed the statistics and wrote the manuscript. GL, SC, and MX performed the sequencing experiments and analyzed the sequencing data. LS submitted the manuscript. All authors contributed to the article and approved the submitted version.

## Conflict of Interest

XL, GL, SC, MX, and LS are employed by the company HaploX Biotechnology, Co., Ltd. The remaining authors declare that the research was conducted in the absence of any commercial or financial relationships that could be construed as a potential conflict of interest.

## Footnotes

1 https://github.com/etal/cnvkit

2 https://www.r-project.org/

## Supplementary Material

FIGURE S1 Correlation of PD-L1 expression level with other markers or patient response or survival. Results of correlation were shown for PD-L1 expression with PFS **(A)**, bTMB **(B)**, ctDNA concentration **(C)**, response **(D)**. The influence of PD-L1 expression (negative or positive) on survival was shown in panel **(E)**, and the influence of PD-L1 expression (<50% or ≥50%)was shown in panel **(F)**. PD-L1 expression level of >1% was considered positive, and PD-L1 expression level of ≥50% was considered high expression. In 15 patients with available PD-L1 expression data, 11 had expression level of >1% and 4 had expression level of ≥50% (Supplementary Table S3).
