# Supplementary figures and images for: ctDNA Concentration, MIKI67 Mutations and Hyper-Progressive Disease Related Gene Mutations Are Prognostic Markers for Camrelizumab and Apatinib Combined Multiline Treatment in Advanced NSCLC
Source: Front Oncol. 2020 Sep 4;10:1706. doi: 10.3389/fonc.2020.01706 (PMC7509428; doi:10.3389/fonc.2020.01706)

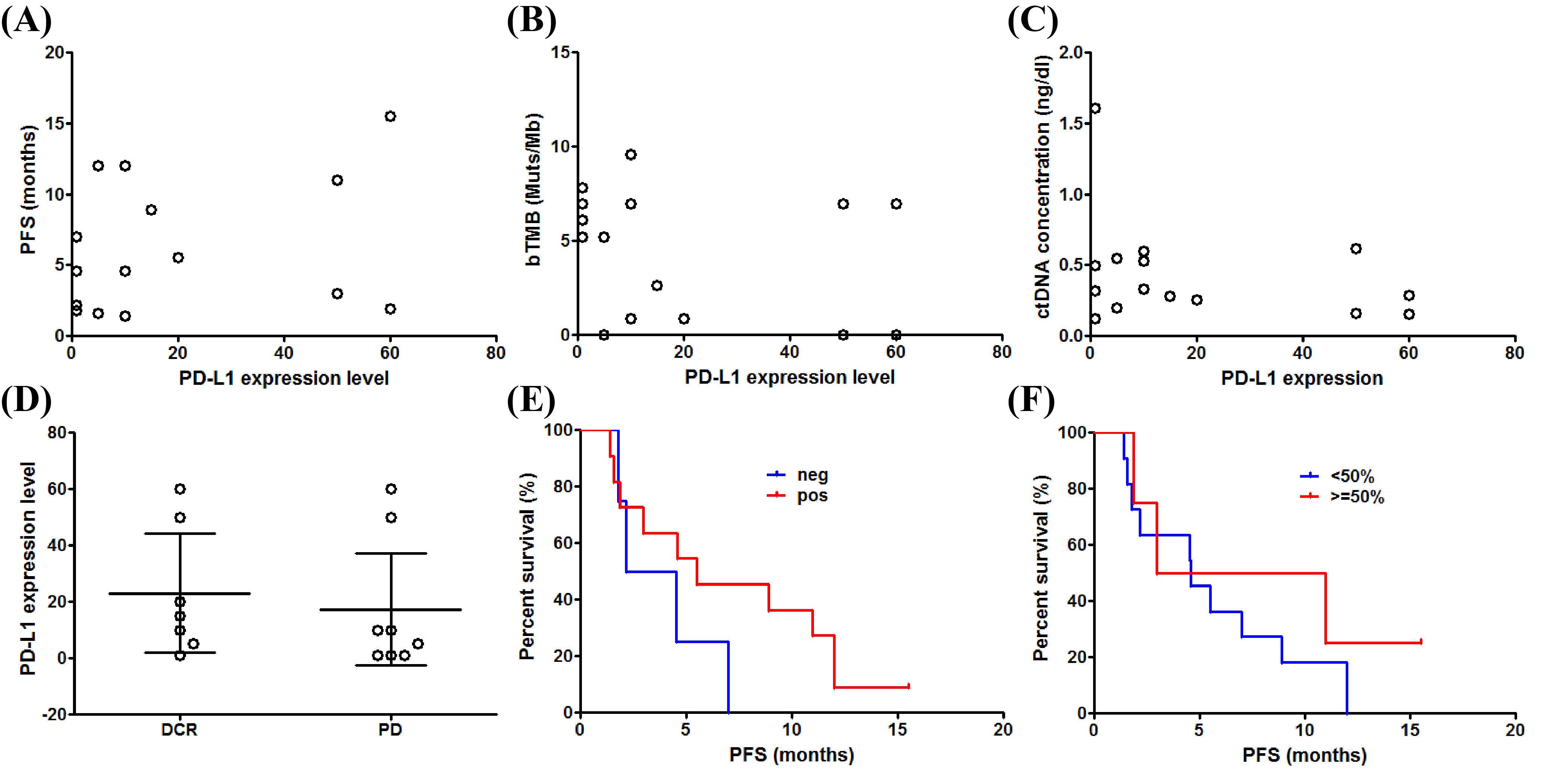

Supplement: FIGURE S1 — Correlation of PD-L1 expression level with other markers or patient response or survival. Results of correlation were shown for PD-L1 expression with PFS (A), bTMB (B), ctDNA concentration (C), response (D). The influence of PD-L1 expression (negative or positive) on survival was shown in panel (E), and the influence of PD-L1 expression (<50% or ≥50%)was shown in panel (F). PD-L1 expression level of >1% was considered positive, and PD-L1 expression level of ≥50% was considered high expression. In 15 patients with available PD-L1 expression data, 11 had expression level of >1% and 4 had expression level of ≥50% (Supplementary Table S3). [file Image_1.TIF]

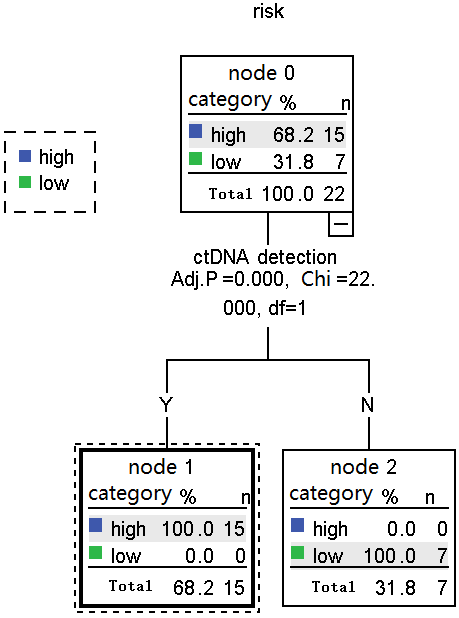

Supplement: Supplementary file 2 [file Image_2.TIF]
